# Meckel’s Diverticulum as the Lead Point of Ileo-ileal Intussusception in a Young Adult: A Case Report

**DOI:** 10.7759/cureus.94506

**Published:** 2025-10-13

**Authors:** Jose A Vergara Torrente, Gerardo E Muñoz Maldonado, Carolina Valencia Sepulveda, Carlos F Sánchez Calderón

**Affiliations:** 1 General Surgery, Hospital Universitario Dr. José Eleuterio González, Monterrey, MEX

**Keywords:** computed tomography (ct ), intussusception, meckel’s diverticulum, small-bowel obstruction, small-bowel resection

## Abstract

Adult intussusception is uncommon and typically arises from a lead point; Meckel’s diverticulum (MD) is a rare cause. Its clinical presentation often overlaps with other etiologies of bowel obstruction, making imaging and intraoperative confirmation decisive.

A 27-year-old woman with no comorbidities presented with seven days of generalized abdominal pain, nausea, vomiting, and absence of canalization of gases and bowel movements. Contrast-enhanced CT revealed an 11 cm ileo-ileal intussusception.

As part of her management and outcome, a laparotomy was performed, which confirmed a 20 cm ileo-ileal intussusception secondary to MD located 60 cm from the ileocecal valve. Segmental resection of the affected ileal segment with end-to-end anastomosis was carried out. The postoperative course was favorable, and the patient was discharged in good condition.

In adults, MD can act as a lead point for intussusception and cause obstruction. Clinical findings are often nonspecific; CT guides diagnosis and surgical planning. Segmental resection is the treatment of choice and, when undertaken promptly, is associated with favorable outcomes.

MD should be included in the differential diagnosis of bowel obstruction in young adults. Clinical suspicion supported by CT and early surgical intervention promotes optimal recovery.

## Introduction

Intussusception occurs when one segment of intestine invaginates into an adjacent segment, producing progressive traction on the bowel wall (“telescoping effect”) [1]. It is common in the pediatric population and may present with the classic triad (abdominal pain, palpable mass, and bleeding), although the triad is often incomplete [1], making it a frequent cause of intestinal obstruction. By contrast, in adults, it is estimated that only about 5% of bowel obstructions are due to intussusception [1].

In children, one of the most frequent causes is Meckel’s diverticulum (MD), a malformation resulting from incomplete involution of the omphalomesenteric duct during embryogenesis, with an approximate prevalence of 2% in the general population [2,3].

In adults, most intestinal intussusceptions arise from an identifiable structural lead point. Across several series, neoplasms predominate as the etiology. Consequently, in clinical practice, management is frequently directed toward surgical resolution [1,3,4].

Herein, we describe the case of a previously healthy woman who developed intestinal obstruction secondary to ileo-ileal intussusception caused by MD [5-7].

## Case presentation

A 27-year-old female patient, without relevant medical history, presented to the emergency department with a seven-day history characterized by moderate, diffuse abdominal pain accompanied by nausea and vomiting of gastric contents. In the three days prior to admission, she developed obstipation (absence of canalization of gases and bowel movements). As the condition progressed, she experienced abdominal distension and increased pain intensity. She denies genitourinary symptoms.

On examination, the neck was unremarkable; chest auscultation revealed preserved vesicular breath sounds without adventitious noises. The abdomen was soft and depressible with mild distension and diffuse tenderness to both superficial and deep palpation, without peritoneal signs; bowel sounds were present and hyperactive. The neurological examination was intact, with no additional pathological findings.

**Figure 1:**
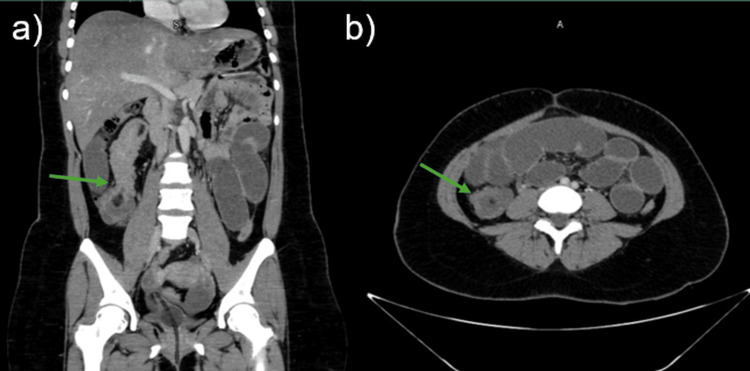
Abdominal computed tomography (a) Coronal view, arrow indicates the intussusception; (b) axial view arrow indicates the target sign.

Laboratory and imaging studies were obtained. Complete blood count revealed leukocytosis of 14,000/µL driven by neutrophils (90%); serum electrolytes showed hypochloremia with chloride at 94 mmol/L; C-reactive protein was 9.4 mg/dL; blood urea nitrogen (BUN) 30 mg/dL; and creatinine 0.8 mg/dL, with no other relevant abnormalities. Contrast-enhanced abdominopelvic computed tomography (CT) demonstrated diffuse dilation of small bowel loops with a “sausage sign” on coronal reconstructions and a “target sign” on axial images, findings suggestive of an ileo-ileal intussusception measuring approximately 11 cm in length (Figure 1).

**Figure 2:**
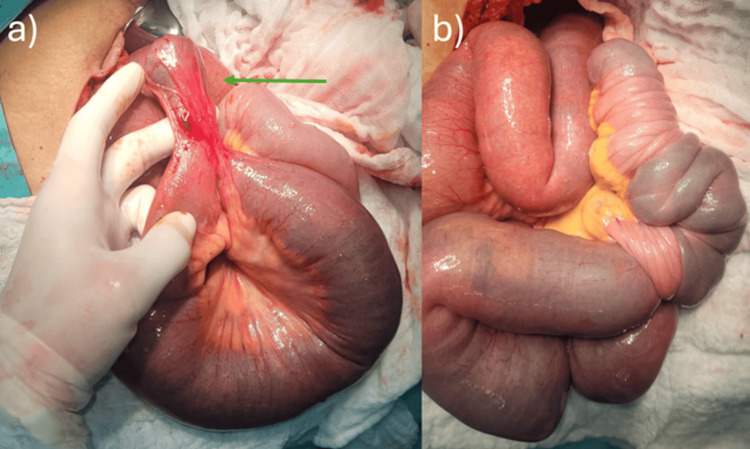
Intraoperative images (a) Arrow indicates the Meckel’s diverticulum; (b) intussusception beginning 60 cm proximal to the ileocecal valve.

Based on these findings, an exploratory laparotomy was performed, confirming a 20-cm ileo-ileal intussusception secondary to a Meckel’s diverticulum located 60 cm proximal to the ileocecal valve (Figure 2). A segmental small-bowel resection with primary end-to-end anastomosis was undertaken, with an anticipated favourable clinical course. Histopathological analysis demonstrated a true diverticulum containing all layers of the intestinal wall, confirming the diagnosis of MD.

During the hospital stay, oral intake was progressively advanced, and the patient was discharged on postoperative day four without complications.

Written informed consent was obtained from the patient for publication of the case and images and ethical approval is not applicable (single patient case report with no intervention beyond the standard of care).

## Discussion

Intussusception can be classified by location as enteroenteric (small bowel), colocolic (colon), ileocolic, and ileocecal; in adults, enteroenteric forms are the most frequent, whereas ileo-ileocolic presentations are less common [1,3,8,9]. In addition, MD shows a higher prevalence in men, with male-to-female ratios of 1.5:1 to 4:1 [8].

In this context, our case of a 27-year-old woman is illustrative because it falls outside the reported sex distribution pattern, underscoring the need to maintain a high index of suspicion in female patients as well.

Adult etiologies are heterogeneous. Neoplastic causes predominate, with a substantial proportion of malignant lesions; among benign lead points, inflammatory processes, MD, and adhesions are notable [4,3]. Obstruction secondary to intussusception due to MD is infrequent, documented only in a minority of patients with this congenital anomaly [2]. In our patient, the seven-day clinical course with progression to constipation, abdominal distension, and increased pain is consistent with the nonspecificity described in the literature for adult presentations, in which symptoms overlap with other causes of bowel obstruction [2-4,8].

CT is the most useful imaging tool, with reported sensitivities ranging from 58% to 100% [3,10]. In this case, CT demonstrated diffuse small-bowel dilation and the classic signs: a “target” sign on axial images and a “sausage-shaped” appearance on longitudinal planes, consistent with an 11 cm ileo-ileal intussusception. These findings correlated intraoperatively with a 20 cm intussusception. This concordance between imaging and surgical findings reinforces the value of CT for diagnostic guidance and surgical planning [3,10].

At surgery, the MD was identified 60 cm from the ileocecal valve, within the 20-100 cm range described in clinical series. Hernández et al. reported a male predominance, in contrast to our case, but a nonspecific presentation and a diverticular location similar to what we observed here [9]. Segmental resection of the involved segment with end-to-end anastomosis was the treatment of choice, in keeping with recommendations for adult intussusception with an identifiable lead point, and it was associated with a favorable postoperative course, as described in contemporary series [3,10].

The principal complications of MD include obstruction, bleeding, and inflammation; recent series report approximate rates of 30%-36%, 25%-30%, and 30%, respectively [2]. In our case, obstruction was the main manifestation, with no evidence of active bleeding or diverticulitis, highlighting that the clinical spectrum of MD is variable and that intussusception should be considered among the differential diagnoses in young adults with bowel obstruction.

Taken together, this case offers three practical messages: presentation in a young woman reminds us that sex distribution does not preclude the diagnosis; the concordance between CT signs and intraoperative findings supports the central role of CT in decision making; and timely segmental resection in the setting of an identifiable lead point is associated with a favorable outcome [2-4,8-10].

## Conclusions

Adult intussusception poses a significant diagnostic challenge owing to its nonspecific clinical presentation, which often overlaps with other causes of bowel obstruction. Although neoplasms constitute the most frequent etiology in this population, clinicians should maintain a broad differential, particularly when CT demonstrates features consistent with intussusception as the underlying cause of obstruction. In this setting, MD should be considered a potential lead point even in young patients without apparent comorbidities.

This case reinforces the importance of early clinical suspicion supported by high-quality imaging and intraoperative confirmation, enabling timely surgical management. Segmental resection of the affected bowel, with primary anastomosis when feasible, is commonly associated with favourable outcomes and a lower risk of complications. In summary, incorporating MD into the differential diagnosis of intestinal obstruction in young adults can streamline evaluation, shorten time to intervention, and improve postoperative recovery.
